# Establishment of a promising vitiligo mouse model for pathogenesis and treatment studies

**DOI:** 10.1186/s13000-024-01520-2

**Published:** 2024-07-03

**Authors:** Ruirui Fan, Jie Gao

**Affiliations:** 1 Department of Pathology, Xiang’an Hospital of Xiamen University, School of Medicine, Xiamen University, Xiamen, China; 2 Department of Dermatology, the First Affiliated Hospital of Chongqing Medical University, Chongqing, China; 3 Department of Pathology, Xiamen Humanity Hospital Fujian Medical University, Xiamen, China

**Keywords:** Vitiligo, Vitiligo animal model, Model construction, Identification

## Abstract

**Aims:**

Vitiligo is a chronic dermatological condition characterized by the progressive loss of melanocytes, for which traditional therapy has shown limited efficacy. This study aimed to establish a vitiligo model with easy operability, high repeatability, and stable depigmentation to provide a foundation for studying the pathogenesis and developing novel therapies for vitiligo.

**Methods:**

(1) Establishing vitiligo model: Firstly, deliver B16F10 cells to the back skin of C57BL/6 J via intradermal injection (day 0), and the CD4 depletion antibody was injected intraperitoneally on day 4 and 10. Secondly, the melanoma was surgically removed on day 12. Thirdly, CD8 antibody was administered intraperitoneally every fourth day till day 30. (2) Identification of vitiligo model: H&E staining, immunohistochemistry, and immunofluorescence were used to detect the melanocytes. The melanin was detected by transmission electron microscopy (TEM), Lillie ferrous sulfate staining and L-DOPA staining.

**Results:**

(1) The back skin and hair began to appear white on day 30. Melanin loss reached peak on day 60; (2) Hematoxylin and eosin (H&E) staining, immunohistochemistry and immunofluorescence results showed melanocytes were reduced. L-DOPA staining, Lillie ferrous sulfate staining and TEM results showed that melanin decreased in the epidermis.

**Conclusion:**

We successfully establishment a vitiligo mouse model which can be more capable to simulate the pathogenesis of human vitiligo and provide an important basis for the study of pathogenesis and therapy of vitiligo.

## Introduction

Vitiligo is an autoimmune skin disease affecting 0.5–2% of the global population, significantly impacting patients' quality of life and causing substantial psychosocial stress [1]. The etiology and pathogenesis of vitiligo remain unknown, with various hypotheses proposed to address its pathogenesis, including roles for innervation, microvascular anomalies, oxidative stress-induced melanocyte degeneration, defects in melanocyte adhesion, autoimmunity, somatic mosaicism, and genetic influences [2]. The autoimmunity theory suggests that self-reactive immune cells attack melanocytes, leading to their death and subsequent loss from the epidermis, resulting in depigmentation [3].

No cure exists for vitiligo. Despite available treatments, many vitiligo patients do not respond well, emphasizing the need for more effective therapies. Animal models play a crucial role in mechanistic studies, and suitable models are essential for studying pathogenesis and treatment approaches [4]. Vitiligo animal models can be classified into spontaneous models and induced models. Compared with the spontaneous vitiligo models, induced vitiligo models can be more capable to simulate the pathogenesis and influencing factors of human vitiligo. None of the existing animal models can completely simulate all the mechanisms of human vitiligo pathogenesis, but each model has its own emphasis and advantages [5].

The vitiligo model induced by chemical decolorization agent has the advantages of low cost and easy operation. However, skin decolorization is mainly due to the loss of melanocytes caused by chemical stress, and the decolorization is unstable, which cannot fully reflect the pathological characteristics of the vitiligo patients [6]. The advantage of melanocyte stress-induced vitiligo model is that the procedure is simple, but the model construction method is not uniform, and the depigmentation effect is unstable [7]. The vitiligo model induced by exogenous T cells requires high technical operation, and the depigmentation effect is unstable [8]. The process of transgenic animal model is complicated, and the cost is high [9]. The model of vitiligo induced by melanocyte detachment does not involve an immune response and is only suitable for the study of vitiligo by non-immune mechanisms [10]. The vitiligo model established by immune induction is simple, high repeatability, and short experiment period. Besides, this vitiligo model does not have skin inflammation, redness and swelling, and can be depigmented for more than 1 year [11].

At present, there are many methods to establish vitiligo models, but most studies do not have sufficient data to confirm that the established models are vitiligo models. In our study, a variety of techniques were employed to validate the developed model by detecting melanocytes and melanin in skin, for example, symptom observation, WOOD UV lamp, ophthalmic slit lamp, H&E staining, immunohistochemistry, immunofluorescence, special staining, and TEM. The vitiligo mouse model we established would better simulate the pathogenesis of human vitiligo and provide an important experimental basis for studying pathogenesis and developing new therapy pathway of vitiligo. Our study work will be a supplement to previous studies and has greater theoretical and practical significance.

## Materials and methods

### Cell culture

B16F10 cells were maintained in DMEM medium (Gibco,USA) supplemented with 10% (v/v) FBS(Gibco,USA) and 1% (v/v) antibiotic–antimycotic(Gibco,USA) at 37 °C in a cell incubator with 5% CO2. The cells were passaged after they covered approximately 80% of the growth surface area of the culture flask.

### Animals and experimental design

In our study, male C57BL/6 mice of age 7 weeks and weight approximately 20 g were used as animal models. C57BL/6 J mice were obtained from Xiamen University Laboratory Animal Center. Animals were housed in Plexiglas boxes (25 × 25 × 15 cm), under standard conditions (temperature: 20 ± 2 °C, humidity: 50 ± 5%, 12 h light– dark cycle, and free access to food and water). Mice were briefly divided into 2 groups: Group 1—Healthy control: Untreated mice (*n* = 3), Group 2—Experimental group: Treated mice (*n* = 3).

### Vitiligo animal model

Day 0: Adjust the B16F10 cells concentration to 1.6 × 10^3^/ μL in the DMEM, which corresponds to the final 2 × 10^5^ B16F10 cells inoculum per 125 μL injection volume. Use an insulin syringe to deliver 125 μL of the B16F10 cells to the back skin via intradermal injection. The angle of intradermal injection should be approximately 10 degrees, slightly bend the needle of the syringe to make the injection angel almost parallel to the skin surface, with the bevel side up. The needle is inserted to a depth of 3 mm. The control group received only the same amount of DMEM intradermal injection. Day 4 and 10: The primary tumor usually forms about a week after the B16F10 cells are implanted. Check the tumor size with a vernier caliper at the intradermal injection site. The CD4 depletion antibodies were injected intraperitoneally on days 4 and 10. Only mice that developed primary tumors were used for further analysis. Spontaneous tumor metastases were not observed. Mice were injected with 10 μg antibodies per g of weight. Antibody preparation was performed at 20 g per mouse. If the number of target mice is N, prepare the amount of CD4 antibody(μg) = 10 μg/g × 20 g/ mice × (N + 1) mice. CD4 antibody was diluted to 1 μg/μL using sterile PBS. CD4 antibody was intraperitoneally injected according to the weight of each mouse at 10 μg/g, stayed for 4 s after injection, and then the needle was withdrawn. Control mice received only the same amount of PBS intraperitoneally. Day 12: Primary melanoma was surgically removed. Skin above the tumor should be resected with the melanoma. Be careful not to cut through the tumor during surgery. Try to completely resect the tumor, for the residual melanoma cells will result in recurrence of the melanoma. Use suture clips to close the wounds and clean the surgical area with gauze. Day 16–28: CD4 antibody was intraperitoneally injected according to the weight of each mouse at 10 μg/g as described before. CD8 antibody was administered every fourth day after the tumor was removed. The immunized mice were daily observed for the appearance of depigmented lesions and survival. We did not witness a single death in animals of any group. Control mice received only the same amount of PBS intraperitoneally. Day 30–60: Melanocytes began to reduce at day 30, and epidermis depigmentation was visually apparent by day 60.

### Identification of vitiligo mouse model

#### Immunohistochemical staining

Immunohistochemical staining was performed to detect the melanoma and melanocytes. Serial sections were cut from the paraffin-embedded blocks. Subsequen-t deparaffinization and rehydration in xylene and graded series of alcohol were performed. The tissue was treated with 3% Triton X-100 (Sigma,USA) for 20 min. The slides were incubated overnight at 4 °C with anti-mouse tyrosinase (TYR) antibody (Santa, USA,1:100), Tyrosinase-associated protein 1(TRP1) antib-ody (Santa, USA,1:100), Melan-A antibody (Santa,USA,1:100),Microphthalmia-associated transcription regulatory factor (MITF, GeneTex,USA,1:150) antibody. A secondary antibody was then added to sections. Normal skin was used as a control group.

#### Immunofluorescence assay

Immunofluorescence staining was performed on skin sections to check the melanocytes. Sections without paraffin were fixed in paraformaldehyde. The primary antibodies used were anti-mouse TYR and MITF. Tissues or cells were incubated overnight at 4 °C with the antibodies. A secondary antibody was then added to sections, followed by incubation at 25 °C for one hour without exposure to light. Finally, Hoechst 33,342 (Sigma-Aldrich) was used to stain cell nuclei for 10 min without light.

#### L-DOPA staining

4 μm thick sections of lesional skin of vitiligo mice model were prepared, and L-DOPA staining was performed to observe the melanocytes and melanin. L-DOPA (10 mM) was added to the tissues and incubated for 3.5 h at 37℃. After incubation was over, the sections were again washed twice with PBS and examined under a microscope.

#### Transmission electron microscopy (TEM)

After skin fixation of vitiligo group and normal group, the carrier copper mesh was left for 1 min at room temperature and the liquid was absorbed by filter paper along the outer side of the copper mesh. Then 20 mL/L phosphotungstic acid solution 30ul was added to the copper mesh. Finally, the filter paper absorbed the phosphotungstic acid solution at 25℃ for 1 min. The copper mesh was baked under incandescent lamp for about 10 min.

#### Lillie ferrous sulfate staining

The skin tissue sections were washed in ddH_2_O and then immersed in ferrous sulfate solution for 1 h, and cleaned with ddH_2_O for 5 times, each time for 2 min. It was soaked in acid potassium ferricyanide solution for 30 min, cleaned by ddH_2_O, immersed in nuclear solid red dye solution for 5 min.

#### Wood UV lamp detection

The back and ear hair of vitiligo mice were removed. Wood UV lamp was used to irradiate the back and ear skin of the vitiligo mice and compared with the control group.

### Statistical analysis

Data are presented as the mean ± SD from three independent experiments. All statistical analyses were performed in SPSS using a t-test, and results with a *p*-value < 0.05 were considered statistically significant.

## Results

### Establishment of vitiligo mouse model

#### The vitiligo model was successfully established

**Fig. 1:**
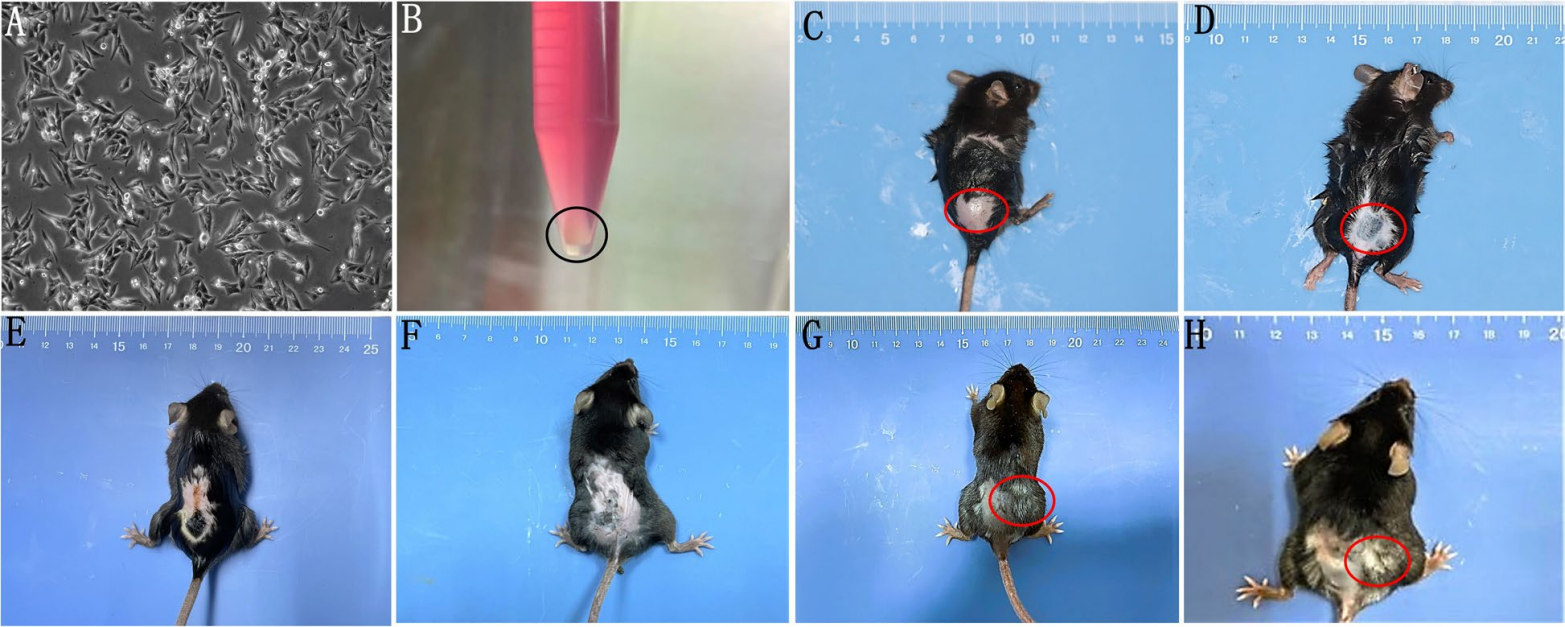
**A**, **B** B16F10 cell. **C** C57BL/6 J mice with intradermal injection of B16F10 cells(D0). **D** The max diameter of the tumor was about 1 cm(D12). **E**, **F** CD8 antibody was injected intraperitoneally on day 16(E),20 and 24(F). **G**, **H** The back skin began to turn pale and dry, and the back hair appeared white on day 30(G) and 60(H)

Deliver B16F10 cells to the back skin via intradermal injection as described in the previous methods. On day 12, the primary tumor was surgically removed, and the max diameter of the tumor was about 1 cm. On day 30 and 60, the back skin began to turn pale and dry, and the back hair began to turn white (Fig. 1).

#### Melanoma was confirmed by H&E staining and immunohistochemistry, and CD4/CD8 + T immune cells infiltration in the tumor was found

**Fig. 2:**
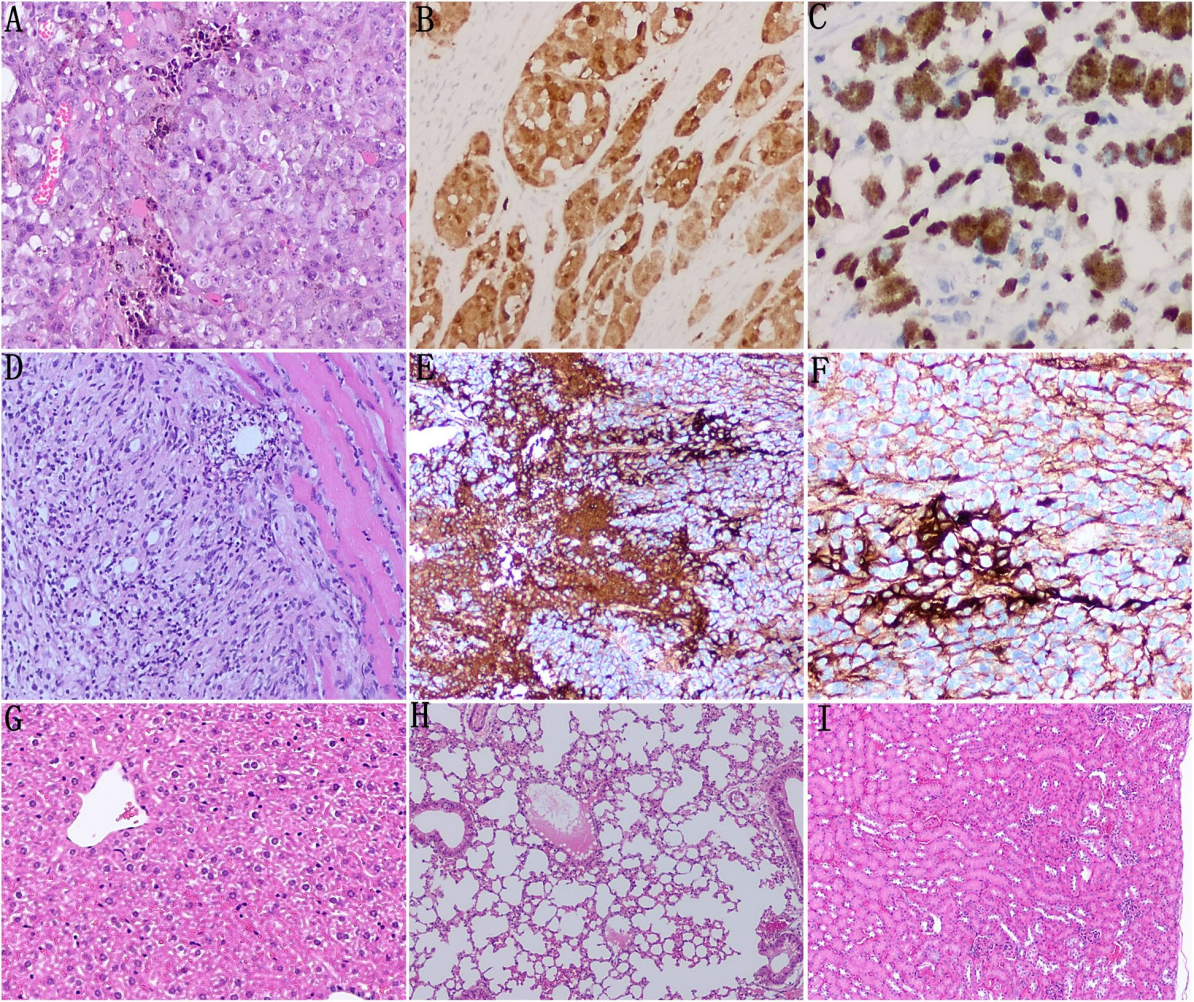
**A** H&E staining showed the histological morphology of melanoma. **B**, **C** Immunohistochemistry results show the tumor expressed Melan-A, and TYR. **D** H&E staining results showed the immune cells infiltration around the tumor. **E**, **F** Immunohistochemistry results showed that the immune cells expressed CD4 and CD8. **G**-**I** No metastasis was found in liver, lung, and kidney by H&E staining

H&E staining and immunohistochemistry results showed the histological morphology and immune markers of melanoma. The infiltration of immune cells in the tumor was found by H&E staining and the immune cells expressed CD4 and CD8 by immunohistochemistry, which confirmed that after the activation of autoimmune reaction in mice, melanocytes in the epidermis were attacked, resulting in the death and shedding of melanocytes. No tumor metastasis was found in liver, lung, and kidney of the experimental group (Fig. 2).

### Identification of vitiligo mouse model

#### Back hair, ear hair, tail skin and cornea of eye in experimental group mice were depigmented

**Fig. 3:**
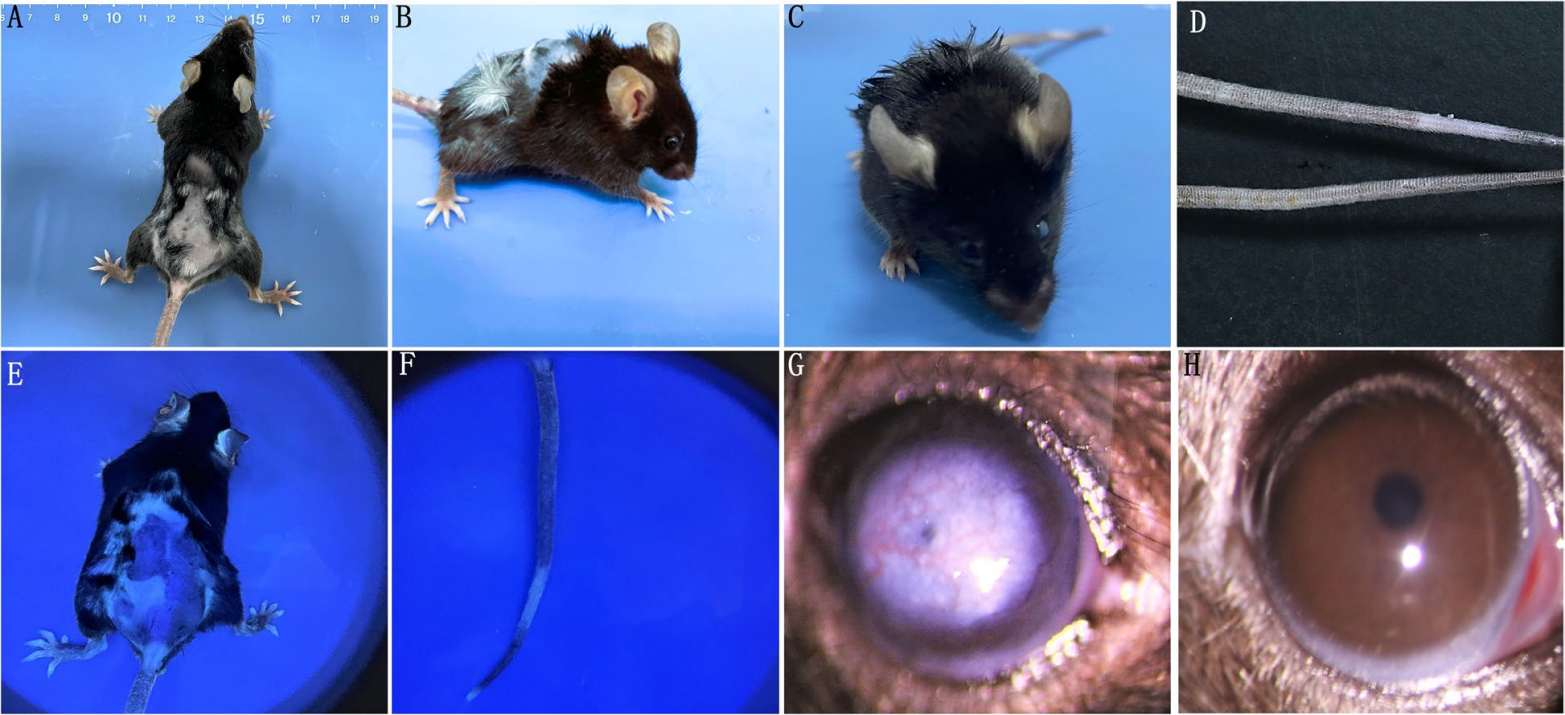
**A**, **B** The back skin and hair turned white in vitiligo model. **C** The ear skin and hair turned white. **D** The pigmentation of the tail skin decreased(upper). **E**, **F** Under WOOD UV lamp, the back skin and tail skin were bright blue. **G** Corneal leukoplakia and vascular congestion were found by ophthalmic slit-lamp. **H** Control group under the ophthalmic slit-lamp

On day 60, the back hair and ear hair became depigmented, and the pigmentation of the tail skin decreased compared with the control group. The back skin and tail skin were bright blue by WOOD UV lamp in the experimental group. Corneal leukoplakia and vascular congestions were found by ophthalmic slit lamp compared with control group (Fig. 3).

#### The number of hair follicles, melanocytes and melanin was reduced.

**Fig. 4:**
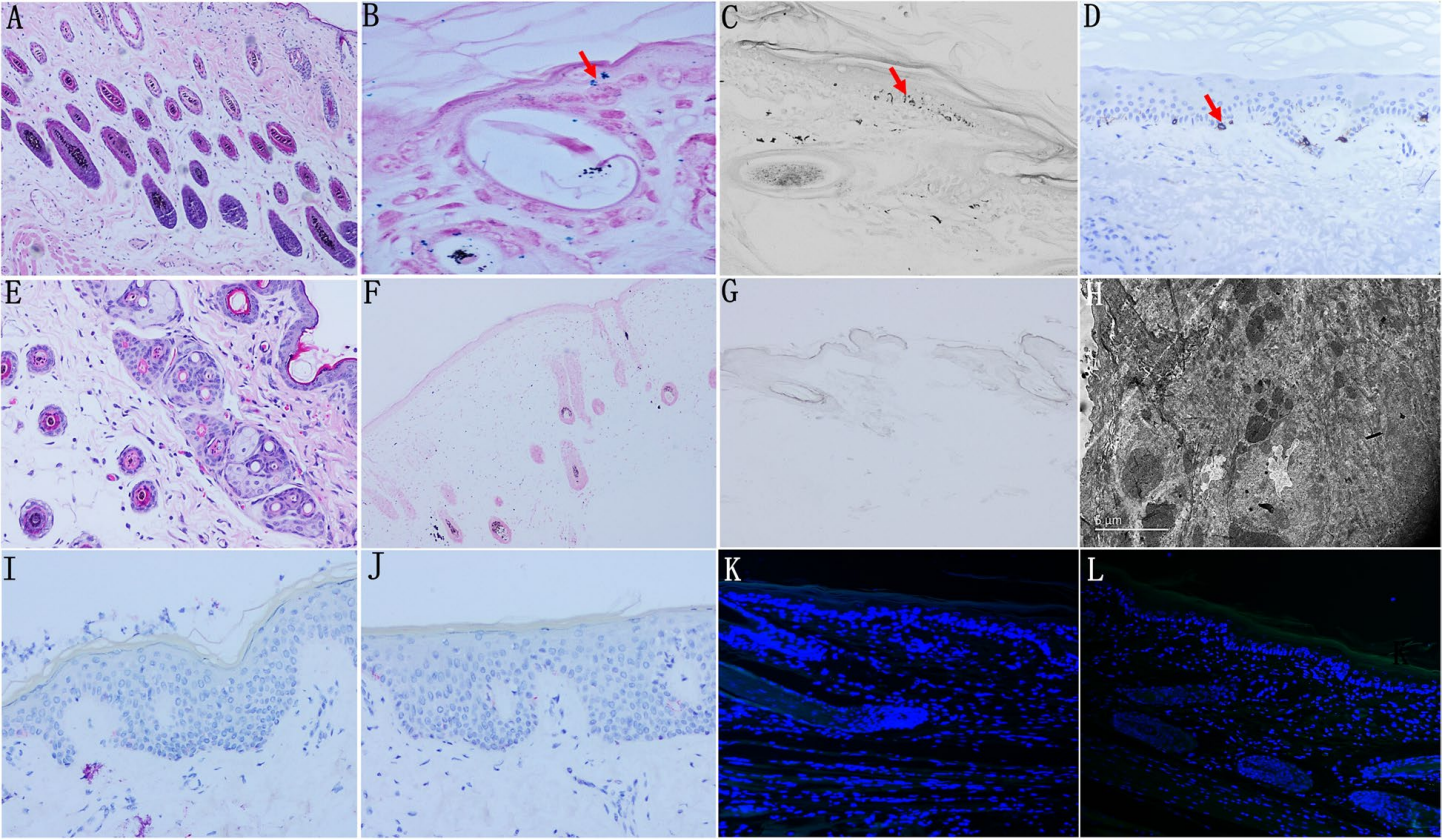
**A** H&E staining showed hair follicles in normal group. **B**, **C** Lillie ferrous sulfateand L-DOPA staining results showed the melanin in normal group. **D** Immunohistochemistry results showed the skin expressed MITF in normal group. **E**–**H** In the experimental group, the amount of hair follicles and melanin was significantly reduced compared with thenormal group by H&E staining, Lillie ferrous sulfate, L-DOPA staining and TEM. **I**-**L** Immunofluorescence and immunohistochemistry results showed the skin expressed no MITF orTYR

H&E staining showed there were some hair follicles in the skin of normal mice, and Lillie ferrous sulfate staining, L-DOPA staining and TEM results showed there were some melanocytes and melanin in skin. But hair follicles were significantly reduced in experimental group by H&E staining. Lillie ferrous sulfate staining, L-DOPA staining and TEM results showed the number of melanin decreased in epidermis compared with the normal group. Immunofluorescence staining and immunohistochemistry results showed the number of melanocytes decreased in skin epidermis compared with the normal group (Fig. 4).

## Discussions

Vitiligo is multi-factorial and has a complex pathogenesis, therefore, studies performed directly in human samples are important, but are illogical. Combining mechanistic findings in animal models with findings reported in humans is a powerful approach to understand vitiligo pathogenesis and to develop novel therapeutic strategies for its treatment. The etiology and pathogenesis of vitiligo have not been fully clarified. The effect of traditional therapy is not obvious, so it is urgent to study the pathogenesis and develop other new therapeutic approaches. In animal experimental research, animal models are widely used in basic and clinical medicine research with their confirmatory research methods. The establishment of a suitable animal model of vitiligo is conducive to further research on the treatment of vitiligo.

Rmadi and Marchioro et al. established a vitiligo mouse model by using chemical decolorization reagents. However, the depigmentation effect is unstable, and cannot fully reflect the pathological characteristics of the vitiligo patients [12]. Al Abadie et al. established a vitiligo mouse model by melanocyte stress inducing. But the model construction method is not uniform, and the depigmentation effect is unstable [13]. Kindl and Zhao et al. established the vitiligo model induced by exogenous T cells requires high technical operation, and the depigmentation effect is unstable [14]. Mukhatayev and Frantz et al. established transgenic vitiligo mouse model. The process of transgenic animal model is complicated, and the cost is high [15]. Hernandez et al. established the vitiligo model induced by melanocyte detachment. The model does not involve an immune response and is only suitable for the study of vitiligo by non-immune mechanisms [16]. None of the existing animal models can completely simulate all the mechanisms of human vitiligo pathogenesis, but each has its own emphasis and advantages. Therefore, establishing a vitiligo animal model with easy operation, high reproducibility and specific epidermal depigmentation can provide an important basis for the treatment and pathogenesis of vitiligo.

We have successfully developed a vitiligo mouse model by melanoma/Treg cell exhaustion induction method which recapitulates key clinical features of vitiligo, including epidermis depigmentation, CD8 + T cell infiltration in skin, and epidermis melanocytes loss. The immune induced vitiligo mouse model was simple, successful and short. Visually apparent epidermis depigmentation occurs 2 months later, and sustainable epidermis depigmentation for more than 1 year. Next, WOOD UV lamp detection, H&E staining, immunohistochemistry, immunofluorescence, L-DOPA staining, Lillie ferrous sulfate staining and TEM were used to identify the mouse model.

In our experiments, our data revealed that animal model we established in vitro was vitiligo mouse model.

To sum up, we successfully establishment a vitiligo mouse model which can be more capable to simulate the pathogenesis of human vitiligo and provide an important basis for the study of the treatment and pathogenesis of vitiligo. Our study work will be a supplement to previous studies and has greater theoretical and practical significance.

## Data Availability

No datasets were generated or analysed during the current study.
